# Vietnamese Public Health Practices in the Advent of the COVID-19 Pandemic: Lessons for Developing Countries

**DOI:** 10.1177/1010539520927266

**Published:** 2020-05

**Authors:** Jeconiah Louis Dreisbach

**Affiliations:** 1 Asian Institute of Management, Makati City, Metro Manila, The Philippines

**Keywords:** public health, coronavirus, COVID-19, Vietnam, developing countries

## Abstract

The 2019 coronavirus disease (COVID-19) presents a great challenge to developing countries with limited access to public health measures in grassroots communities. The World Health Organization lauded the Vietnamese government for its proactive and steady investment in health facilities that mitigate the risk of the infectious disease in Vietnam. This short communication presents cases that could benchmark public health policies in developing countries.

Vietnam reported its first two cases of the 2019 coronavirus disease (COVID-19) on January 23, 2020, which came from two Chinese nationals who visited Ho Chi Minh City for the Lunar New Year holiday festivities. By January 28, 2020, public health experts were immediately able to trace the importation and transmission of the disease in the country from the first cases as published in the *New England Journal of Medicine*.^1^ The following case that emerged came from a Vietnamese businesswoman who returned to Vietnam on January 17, 2020, after a 2-month business trip in Wuhan. This was considered to be the first case of a Vietnamese citizen being infected by the disease. A comprehensive case study on the Vietnamese woman published by the *Lancet Infectious Diseases*^2^ mentioned that “individuals who had substantial contact with the patient and her two colleagues were quarantined and all accommodations and transit methods were decontaminated.”

As the number of cases rose to six on January 1, 2020, the Vietnamese government declared that the country was under an epidemic. On February 13, the Vietnamese health ministry ordered the Son Loi municipality, a town near the capital Hanoi, to be placed on lockdown as cases rose in the area. By January 25, 2020, its government declared that all 16 confirmed coronavirus cases had been cured and discharged from the hospital. The World Health Organization (WHO) representative in Vietnam Kidong Park lauded this success the proactive public health practices of the government at its early stages by “intensifying surveillance, enhancing laboratory testing, ensuring infection prevention, and control and case management in health care facilities, clear risk communication message, and multisectoral collaboration.”^3^

On March 12, 2020, however, the Vietnamese Ministry of Health reported that 31 more cases emerged with the latest patients testing positive after coming from flights in Europe. The WHO announced the day before that they consider Europe to be the epicenter of the COVID-19 pandemic. On tracing this development, the country’s flag carrier Vietnam Airlines immediately reduced its flight frequencies between Vietnam and the United Kingdom, France, and Germany as a preventive measure. They also stopped issuing visas on citizens from Italy and South Korea, two of the most infected countries affected by COVID-19, to ensure the public health safety of the Vietnamese people.^4^

Vietnamese Prime Minister Nguyen Xuan Phuc called for the immediate response on the confirmed COVID-19 cases in Vietnam by offering free testing and treatment to those who were found positive, requiring a health declaration from all domestic and international travelers, wearing facemasks in public spaces, and banning the entry of individuals with suspected cases.^5^

In an innovative effort to mitigate the rise of coronavirus cases in the country, the newly formed Vietnamese Steering Committee for COVID-19 Prevention and Control launched a mobile application wherein citizens can be educated about the diseases and its preventive measures.^6^ Moreover, they can be able to frequently update their health conditions and report suspected cases in grassroots communities in the mobile app and identify which areas need immediate action.
